# Enhanced Cellulose Extraction from Banana Pseudostem Waste: A Comparative Analysis Using Chemical Methods Assisted by Conventional and Focused Ultrasound

**DOI:** 10.3390/polym16192785

**Published:** 2024-09-30

**Authors:** Alba N. Ardila A., Erasmo Arriola-Villaseñor, Efraín Enrique Villegas González, Hegnny Estefanía González Guerrero, José Alfredo Hernández-Maldonado, Eduart Gutiérrez-Pineda, Cristian C. Villa

**Affiliations:** 1 Research Group on Environmental Catalysis and Renewable Energies (CAMER), Faculty of Sciences and Education, Politécnico Colombiano Jaime Isaza Cadavid, Medellín PC 050022, Colombia; erasmoarriola@elpoli.edu.co (E.A.-V.); efrainvillegas@elpoli.edu.co (E.E.V.G.);; 2 Unidad Profesional Interdisciplinaria de Ingeniería Campus Guanajuato del Instituto Politécnico Nacional—UPIIG, Av. Mineral de Valenciana 200, Col. Fraccionamiento Industrial Puerto, 36275 Silao, Guanajuato, Mexico; 3 Escuela de Ciencias Básicas, Tecnología e Ingeniería (ECBTI), Universidad Nacional Abierta y a Distancia (UNAD), 680001 Bucaramanga, Santander PC 680002, Colombia; eduart.gutierrez@unad.edu.co; 4 Agro-Food Research and Development Laboratory (LIDA), UNAD Universidad Nacional Abierta y a Distancia-José Celestino Mutis Campus, Bogotá PC 111511, Colombia; 5 Chemistry Program, Faculty of Basic Sciences and Technologies, Universidad de Quindío, Carrera 15 Calle 12n, Armenia, Quindío PC 630001, Colombia

**Keywords:** banana pseudostem waste, chemical treatment, conventional ultrasound treatment, focused ultrasound treatment, cellulose

## Abstract

This study investigates the effectiveness of various chemical methods, both ultrasound-assisted and non-assisted, for extracting cellulose from banana pseudostem (BPS) waste, comparing the results with commercial pine and eucalyptus cellulose fibers. Delignification treatments with NaOH (25% and 30%) and H_2_O_2_ (8%) were evaluated, applied with both conventional and focused sonication. Ultrasound-assisted methods, particularly with NaOH, achieved cellulose percentages as high as 99.5%. X-ray diffraction (XRD) analysis revealed that NaOH treatments significantly increased the cellulose crystallinity index, reaching up to 67.9%, surpassing commercial fibers. Scanning electron microscopy (SEM) results showed that NaOH treatments, especially at 30%, improved fiber morphology and exposure. Thermogravimetric analysis (TGA) indicated that methods using NaOH and focused sonication enhanced the thermal stability of the cellulose. Compared to commercial fibers, some samples obtained with the proposed methods demonstrated higher purity, yield, and thermal stability, highlighting the effectiveness of ultrasound-assisted and NaOH methods.

## 1. Introduction

Cellulose is the most abundant biopolymer on Earth and has a wide range of industrial applications, from paper production to the synthesis of advanced biocomposites [1,2,3]. The growing demand for cellulose across various fields has driven the search for more efficient and sustainable methods for its extraction from diverse agro-industrial residues as biomass sources. Research in this area is crucial to improving cellulose extraction processes and meeting industry needs while minimizing environmental impact [4,5]. The use of BPS residues as an alternative and cost-effective source for cellulose extraction offers numerous advantages due to its significant polysaccharide content compared to other biomass sources. Various studies have demonstrated that BPS contains a cellulose percentage ranging from 20% to 44%, making it a competitive, sustainable, abundant, and economical source for cellulose extraction relative to other plant raw materials [6,7,8]. Moreover, BPS represents approximately 12% of the total weight of the banana plant, and upon harvest, generates a substantial amount of agro-industrial waste [7,8]. Utilizing this waste, which would otherwise be discarded, not only enables efficient and sustainable production but also promotes more rational and eco-friendly management of agricultural resources.

Numerous studies have been developed focusing on exploring processes for cellulose extraction from banana residues. The most commonly employed methods are mechanical, chemical, and enzymatic [8,9,10,11,12,13,14,15,16], which can be used independently or in combination [6,7,14,17,18]. In chemical processes, extractions have been carried out with reagents such as sulfuric acid, bisulfite, sodium hydroxide, potassium hydroxide, sulfates, chlorine, nitric acid, hydrogen peroxide, and oxidation with the TEMPO reagent [16,19], among others. Some research reveals that acid treatments have low solid recovery, indicating high solubilization of the material; however, yields of up to 82% have been achieved using 25% sulfuric acid, resulting in high cellulose content compared to treatments with hydrogen peroxide. Nevertheless, this pretreatment is very effective in completely removing hemicellulose content, which allows for greater accessibility to cellulose in the lignocellulosic material [17]. Severe acid concentrations (H_2_SO_4_) up to 25% can result in cellulose concentrations of 66.28%, 3.97% hemicellulose, and 31.15% lignin, compared to other pretreatments, indicating a significant decrease in cellulose, which suggests degradation of cellulose in these severe treatments [20,21,22]. Alkali pretreatment behaves very similarly to acid pretreatment, with the difference that it can achieve yields of 85% using 25% sodium hydroxide.

On the other hand, hydrogen peroxide treatments completely eliminate hemicellulose and have yielded up to 74% cellulose [17]. Treatments with 25% sodium hydroxide and 8% hydrogen peroxide increase the concentration by approximately 75% compared to acid pretreatment. This indicates that these two pretreatments are more efficient for extracting high cellulose content from biomass derived from banana residues [20]. These studies highlight the feasibility of using BPS residues not only as a sustainable source of cellulose but also as a cost-effective solution for the advanced materials industry, thereby reducing the environmental impact of agro-industrial waste, which can represent up to 30% of the waste generated in banana production. In this context, the choice of sodium hydroxide (NaOH) and hydrogen peroxide (H_2_O_2_) as delignification agents is due to their proven effectiveness in extracting cellulose with high purity content. Previous studies have shown that both reagents are capable of efficiently removing lignin, resulting in a higher concentration of cellulose [13]. For instance, research has indicated that the use of NaOH facilitates the removal of hemicellulose and lignin, while H_2_O_2_ is effective for the oxidation and removal of residual lignin, thus improving the quality of the cellulose obtained [15,17,23].

However, in recent years, the integration of chemical methods with ultrasound has been demonstrated to be a promising technique that can further enhance the efficiency of cellulose extraction [24,25,26]. The ultrasonic technique for isolating cellulose in its various structures has emerged as a promising method and has been the subject of numerous studies [12,13,18,26,27,28,29,30,31,32,33,34,35]. This method leverages ultrasonic energy, which is transferred to cellulose chains through a phenomenon known as cavitation. Cavitation involves the formation, growth, and violent collapse of cavities in water. The energy generated by cavitation, ranging from 10 to 100 kJ/mol, corresponds to the energy scale of hydrogen bonds. This ultrasonic impact is capable of gradually disintegrating micron-sized cellulose fibers into nanofibers, as ultrasound can increase the penetration of reagents into the biomass and accelerate chemical reactions, potentially reducing the time and amount of reagent required [21,23,24,25,33,36,37,38,39].

Even so, although some studies have been conducted on ultrasound-assisted cellulose extraction [12,31], there is a lack of detailed comparative analyses between different sonication methods, specifically between the use of conventional ultrasound (ultrasonic bath) and focused ultrasound (ultrasonic probe). This research aims to fill that gap by evaluating the differences and similarities between these two ultrasound approaches. Another critical aspect that our study addresses, and which is often not reported in previous research, is the overall yield of cellulose extraction. These data are essential for subsequent evaluations of the economic and technical feasibility of extraction methods. By reporting the yields, significant added value is provided to the present research, offering a more comprehensive and practical evaluation of the methods used. This information is vital for other research and for the subsequent planning of the industrial implementation of these processes.

The objective of this research is to conduct a detailed comparative study of different chemical methods for cellulose extraction assisted by conventional and focused ultrasound, using BPS residues as a biomass source. We aim to evaluate these methods in terms of yield, the quality of the cellulose obtained, and process efficiency. Additionally, we intend to provide useful data that include detailed yields and crystallinity analysis, which are often not reported in similar studies. The purpose is to advance the development of more sustainable and efficient processes for cellulose extraction, thereby contributing to valorizing agricultural waste and reducing environmental impact, while also supporting rural communities through new economic opportunities.

The extraction of cellulose fibers from banana pseudostem waste presents an environmentally friendly alternative for reducing agricultural residues. A key advantage of our approach is the use of ultrasound, which significantly decreases the reliance on chemical agents. This not only reduces the overall consumption of chemicals but also minimizes the generation of hazardous waste. Any waste produced during the process is easily treatable through neutralization, ensuring that its environmental impact remains low.

Moreover, the liquid phase from the alkaline treatments contains a high concentration of hemicellulose. This valuable component can be recovered through acidification and precipitation using ethanol, a process we are currently implementing in other research efforts. By recovering hemicellulose, we further contribute to the circular economy, enhancing the sustainability of the cellulose extraction process and reducing the environmental footprint compared to traditional methods.

## 2. Methodology (Materials and Methods)

### 2.1. Collection and Preparation of Raw Material

The collection of pseudostems from the Dominico banana (*Musa* spp.) was carried out in the El Barro area, Girardota municipality, Antioquia department. The pseudostems were cut into slices and sun-dried for 120 h to remove part of the moisture from the material. Subsequently, they were dried by convection in an electric oven at 50 °C for 72 h to remove all the water. Next, with the help of scissors, the material was cut into pieces 2 cm wide by 5 cm long and then ground in a blade mill until a fine powder was obtained. Finally, the ground biomass was sieved through an ASTM mesh 80 (180 µm) and stored in plastic bags for further treatment and physicochemical analysis. The material resulting from this stage will be referred to hereafter as GDB

### 2.2. Cellulose Extraction by Chemical and Ultrasound-Assisted Chemical Procedures

For all chemical procedures, a ratio of 1 mL of solution (NaOH or H_2_O_2_) per 0.1 g of biomass was used. Different methods were employed to extract cellulose from dry powdered biomass, using both conventional chemical procedures and ultrasound-assisted methods.

### 2.3. Conventional Chemical Procedures

Alkaline Hydrolysis (CE-Na25 and CE-Na30): GDB was treated with NaOH (25% and 30%) under stirring in an Ultraturrax (Heidolp RZR2021, Heidolph, Schwabach, Germany) at 550 rpm for 2 h at room temperature. The samples were then allowed to stand at room temperature for 12 h, followed by autoclaving at 120 °C and 20 psi for 90 min. Subsequently, the samples were cooled, filtered, washed with deionized water until a pH of 7.0 was achieved in the wash water, and dried at 60 °C for 12 h.

Oxidation with 8% H_2_O_2_ (CE-H08): GDB was mixed with 8% H_2_O_2_ under stirring in an Ultraturrax for 2 h at 550 rpm and room temperature. After a 12 h resting period, the samples were filtered, washed, and dried under the same conditions applied in the alkaline hydrolysis.

### 2.4. Ultrasound-Assisted Chemical Procedures

Alkaline Hydrolysis with Conventional Sonication (CU-Na25 and CU-Na30): GDB samples were treated with NaOH (25% and 30%) under conventional sonication at 40 °C and 300 W for 1 h. The samples were then filtered, washed, and dried under the same conditions applied in the alkaline hydrolysis.

Oxidation with 8% H_2_O_2_ with Conventional Sonication (CU-H08): GDB was mixed with 8% H_2_O_2_ under sonication at 40 °C and 300 W for 1 h. The samples were filtered, washed, and dried under the same conditions described above.

Alkaline Hydrolysis with Focused Sonication (FU-Na25 and FU-Na30): GDB samples were treated with NaOH (25% and 30%) under focused sonication (ultrasonic probe) using a Hielscher ultrasonic processor (Teltow, Germnay) at 200 W and 26 kHz with a UP200St-T transducer and 40% sonication amplitude for 15 min at temperatures between 30 and 40 °C.

H_2_O_2_ 8% with Focused Sonication (FU-H08): GDB was mixed with 8% H_2_O_2_ using a Hielscher ultrasonic processor at 200 W and 26 kHz with UP200St-T transducer and 40% sonication amplitude for 15 min at temperatures between 30 and 40 °C.

### 2.5. Bleaching Process

All samples were subjected to bleaching using a conventional peroxide alkaline process. A specific mass of the obtained material was placed in cylindrical glass reactors. Hydrogen peroxide at 10% was added drop by drop until the sample was completely wetted. The pH was adjusted to 11 by adding drops of 8% NaOH. The mixture was left to stand at room temperature for 2 h to facilitate the bleaching process. Finally, the samples were cooled, filtered, washed with deionized water until a pH of 7.0 was achieved in the wash water, and dried at 60 °C for 12 h. To make comparisons with the samples obtained from the previously described extraction and bleaching methods, commercial short eucalyptus fibers (ESCF) and long pine fibers (PLCF), commonly used in the paper industry, were also characterized. Table 1 shows all the samples obtained according to the different evaluated procedures.

### 2.6. Chemical Composition and Yield

The quantification of extractives and chemical composition was carried out using the gravimetric method, employing a Soxhlet extraction setup. Approximately 5 g of each sample was wrapped in filter paper within a cellulose thimble, which was then placed inside a Soxhlet chamber. This chamber was connected to a 500 mL flask containing 270 mL of deionized water; each sample was analyzed in duplicate. The entire setup was connected to a condenser and refluxed for a period of 8 h. Subsequently, the solvent was recovered via rotary evaporation, and the resulting extractives were dried at 105 °C in an oven until reaching constant weight. This process was repeated using USP-grade ethanol instead of deionized water. Following the extraction of extractives, the chemical composition of the samples was determined. Firstly, the amount of extractives present in water and ethanol was calculated, following the protocol established by the NREL/TP-510-42619 standard [40,41]. Lignin was quantified according to the stipulations of the NREL/TP-510-42618 standard [41]. To complete the determination of chemical composition, holocellulose and cellulose were quantified using the ASTM D-1104 method [42].

### 2.7. X-ray Diffraction Analysis (XRD)

X-ray diffraction analysis was performed using X (DRX) Malvern-PANalytical—Empyean 2012, Malvern, UK, Cu Ka radiation (5avelength 1.541874 Å), operating at 40 kV and 40 mA. The diffraction spectra were logged in 2θ ranging from 15 to 70°, having a scan interval of 0.05°/min and a rate of 1°/min, respectively. The relative crystallinity index (RCI) was estimated with Equation (1). The crystalline and amorphous peaks were deconvoluted (using peak fit Gaussian function) for estimation of area under the peak of the crystalline region (CR) and the amorphous regions (AR).

(1) $$Relative\;Cristallinity\;Index\;(\%)=\frac{I_{CC}}{\left(I_{CC}+I_{CA}\right)}\;\times \;100$$

where *I_CC_* is the intensity peak of the crystalline cellulose, *I_CA_* is the intensity peak of the amorphous cellulose, and *I_Total_* is the total area under the XRD peaks (*I_Total_* = *I_CC_* + *I_CA_*).

### 2.8. Scanning Electron Microscopy (SEM)

The surface micrographs of extracted cellulose were obtained using a scanning electron microscope (JEOL FE-JSM-7100F, Tokyo, Japan). The sample surface was coated with gold by vacuum sputter coater. The images were taken at an accelerating potential of 15 kV in different magnifications. The morphological characterization of the fibers was carried out using scanning electron microscopy (SEM). Measurements were performed with ImageJ software, version 1.8.0. For each sample, two specimens were analyzed, and an average of five micrographs per specimen were used to ensure accurate representation. The number of fibers with similar dimensions within a specified range was counted across these micrographs to obtain the statistical data presented. Additional examples of fibers and their respective measurements, as obtained through the software, are provided in the Supplementary Material for further reference.

### 2.9. Thermogravimetric Analysis (TGA)

The TA Instruments (TGA Q500, New Castle, DE, USA) analyzer was used to perform TGA-DTA analyses. Samples (500 mg) were positioned in a porcelain crucible and heated under the N_2_ atmosphere (flow rate of 100 mL/min) at 10 °C/min from 30 °C up to 500 °C.

## 3. Results and Discussion

### 3.1. Chemical Composition and Yield

The results in Table 2 show the main chemical composition and yield of the dry powdered biomass obtained from untreated BPS residues, as well as the results for the cellulose samples obtained using the different applied methods. After performing the physical treatment to obtain the dry powdered biomass (GDB), the yield was 29.5%, with a lignin content of 30.06%, cellulose content of 41.96%, hemicellulose content of 18.16%, and holocellulose content of 59.22%. These results are consistent with those reported in the literature by other researchers in similar studies [6,10,43,44]. It is relevant to note that possible variations in chemical composition may be attributed to the diversity of BPS species and the geographical and soil conditions where they are cultivated.

In general, ultrasonic treatment (both conventional and focused) has a positive effect on cellulose yields. According to Chagas et al. [12], this can be explained by the acoustic cavitation induced by ultrasound. Cavitation generates heat and promotes the emission of excited species, which then facilitates the relaxation of the biomass surface and causes bond breakage, primarily in regions with defects in the crystalline lattice.

Analysis of the obtained results revealed that all methods implemented for cellulose extraction successfully removed lignin completely, demonstrating significant efficiency in this regard. However, there are notable differences in the cellulose content achieved with each method. The CE-Na25, CE-Na30, CU-Na25, CU-Na30, and FU-Na30 samples exhibited remarkably high cellulose percentages, with some exceeding 90%. On the other hand, ultrasound-assisted methods, as observed in the CU-H08 and FU-H08 samples, also showed high cellulose contents, although slightly lower than those obtained with the non-ultrasound-assisted methods mentioned previously.

In the comparative analysis of the effects of different delignification procedures on cellulose yield, it was observed that concentrations of NaOH at 25% and 30% produced slightly different yields, with the latter being superior to those obtained with H_2_O_2_ at 8%. Additionally, it was found that the application of conventional sonication with both agents resulted in a significant increase in cellulose yields. In contrast, the implementation of focused sonication led to a reduction in cellulose percentages with all delignification agents [17].

In general terms, it is observed that cellulose yield varies slightly between methods, with values ranging from 60.50% to 99.50%. This wide range reflects the significant influence of extraction processes on cellulose recovery efficiency. Comparing the cellulose percentages obtained using methods employing 8% H_2_O_2_ versus those using NaOH at 25% and 30%, a notable difference is observed. The results indicate that alkaline methods, specifically those involving NaOH, yield significantly higher cellulose percentages compared to methods using H_2_O_2_. In particular, the cellulose percentages obtained with H_2_O_2_ methods range from 60% to 68%, while those with NaOH generally exceed 75%. These differences in cellulose yields can be attributed to several factors. First, the use of NaOH, a strong alkaline agent, may lead to better dissolution of non-cellulosic components present in BPS residues, resulting in a higher quantity and purity of the obtained cellulose. Additionally, the alkaline action may facilitate greater breaking of intermolecular bonds in the pseudostem structure, thereby promoting increased cellulose release. On the other hand, while H_2_O_2_ is an effective oxidant, its nature may not be as effective as NaOH in degrading non-cellulosic components and depolymerizing cellulose.

However, it is important to note an exception: in alkaline methods incorporating focused sonication, the resulting cellulose percentages are lower, ranging from 65% to 80%. This difference raises questions about the impact of focused sonication on cellulose extraction efficiency. This variation in results could be attributed to the complex and potentially additive interaction between focused sonication and the chemical agents used. While sonication can facilitate the disintegration of the cell wall, which is mainly composed of lignin, and enhance cellulose release [38], the effect of focused sonication might be counterproductive due to prolonged exposure, creating extreme conditions of mechanical energy and cavitation that could lead to partial degradation of the cellulose [26]. During focused sonication, ultrasonic waves generate cavitation bubbles in the liquid medium, which collapse violently, generating high local temperatures and pressures. This phenomenon may cause the rupture of cellulose chains, thereby reducing its content in the biomass. Moreover, the increased energy generated during focused sonication might also facilitate the dissolution of cellulose in the extraction medium, thereby decreasing its concentration in the solid biomass. Therefore, the combination of focused sonication with alkaline agents could lead to lower cellulose percentages compared to conventional alkaline methods. This observation highlights the importance of considering both mechanical and chemical aspects in the design of cellulose extraction processes to optimize yield and purity of the final product.

In contrast to the low cellulose percentage obtained with ultrasonic probes, it is observed that applying methods with H_2_O_2_ achieves higher yields in the final material. For all methods, whether conventional or sonication-assisted, higher yield percentages are recorded, ranging from 45% to 49% with H_2_O_2_, compared to the use of NaOH, where yields fluctuate between 12% and 20%. The higher yield percentages and lower cellulose percentages obtained with H_2_O_2_ compared to NaOH can be attributed to the fundamental differences in the action mechanisms of these chemical agents. Therefore, although H_2_O_2_ may lead to higher yields in terms of the final mass of the recovered material, the percentage of cellulose in this material may be lower due to the presence of other residual components. H_2_O_2_ is an oxidizing agent that primarily acts on the lignocellulosic components present in the pseudostem residues. During the extraction process, H_2_O_2_ decomposes lignin, a substance that coats and cements cellulose fibers, thereby facilitating their separation and release. Nevertheless, this lignin decomposition process may be partial and selective, resulting in a less pure cellulose extraction with a higher presence of unwanted lignocellulosic residues in the final product.

These results suggest that the use of H_2_O_2_ as the primary chemical agent may be more effective in terms of cellulose extraction performance. This can be explained by the ability of the H_2_O_2_ to decompose and remove non-cellulosic materials present in pseudostem more efficiently than NaOH. Additionally, the oxidative action of H_2_O_2_ may contribute to the depolymerization of cellulose, facilitating its release and extraction to a greater extent. Therefore, the preference for using H_2_O_2_ in these methods may be justified by its capacity to maximize cellulose extraction yield, thereby offering a significant advantage in the production of cellulosic materials from pseudostem residues. When comparing conventional chemical extraction methods with those assisted by sonication, it is noted that sonication-assisted chemical procedures generally exhibit higher overall yields for cellulose extraction. Specifically, extraction with 8% H_2_O_2_ assisted by focused sonication (FU-H08) and conventional sonication (CU-H08) results in the highest observed yields. The latter method demonstrates a notably superior yield compared to the others, reaching 33.58%. Similar results were obtained by Guo Xuxia et al. [26], who reported average yields for obtaining cellulose nanocrystals through acid hydrolysis assisted by ultrasound of 52.8%, 65.3%, and 71.0% for hydrolysis periods of 45 min, 90 min, and 120 min, respectively, compared to 18.3%, 59.7%, and 62.5% for the same times without ultrasound.

Comparing the results obtained from different samples with those of the commercial fibers used as control samples (ESCF and PLCF), it is evident that several of the applied methods achieved cellulose percentages that are similar to or even exceed those of the commercial fibers. For instance, the CE-Na30, CU-Na25, and CU-Na30 samples exhibit cellulose contents of 90.32%, 89.52%, and 99.50%, respectively, surpassing the cellulose percentages of the commercial fibers (77.52% and 77.15%). This finding highlights the effectiveness of the chemical methods used, particularly those assisted by ultrasound and with sodium hydroxide, in improving cellulose yield compared to commercial fibers. This not only underscores the potential of these methods for applications in the paper industry but also the possibility of optimizing the cellulose extraction process for higher yields and purity.

In order to complement the data on the surface chemical composition of the fibers, diffuse reflectance infrared Fourier transform (DRIFT) analyses were performed. The DRIFT spectra, along with their corresponding explanations and comparisons for the different synthesis methods employed, are provided in the Supplementary Material (Figures S1–S12). This additional information offers further insight into the chemical characteristics of the fibers across the various treatments.

### 3.2. X-ray Diffraction Analysis (XRD)

**Figure 1:**
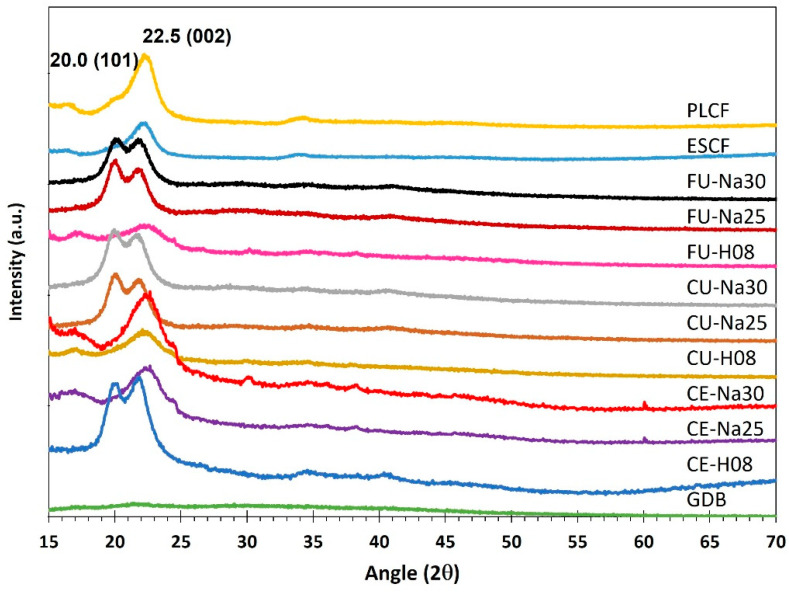
X-ray diffraction pattern of the untreated biomass and the samples obtained with the different methods applied.

The diffractograms obtained reveal significant differences between the various samples, particularly in the appearance of peaks at 2θ = 20.0° and 2θ = 22.5°, corresponding to cellulose types I and II, respectively (Figure 1) [21]. Crystallinity index of the untreated sample (GDB) is remarkably low, with a value of 8.2%. This is due to the amorphous nature of the raw cellulose in the banana pseudostem, which had not undergone any purification or structural enhancement processes. Samples treated with 8% H_2_O_2_, both with conventional ultrasound (CU-H08) and focused ultrasound (FU-H08), exhibit both peaks, suggesting the presence of mixtures of cellulose types I and II. This indicates that treatment with H_2_O_2_ preserves some characteristics of native cellulose (type I) while inducing the formation of regenerated cellulose (type II). The presence of both peaks may be due to a less aggressive oxidation process, which allows for the maintenance of the original crystalline structure while promoting partial transformation to cellulose type II.

On the other hand, in samples treated with 25% and 30% NaOH, only the peak at 2θ = 22.5° (002), corresponding to cellulose type II, is observed. This finding is consistent with the report by Nascimento et al. (2023), as this type of cellulose is one of the primary polymorphic crystalline structures of cellulose [9]. This suggests that alkaline treatment is more effective in completely transforming native cellulose into regenerated cellulose. The absence of the peak at 2θ = 20.0° in these samples indicates that NaOH induces dissolution and subsequent recrystallization of cellulose, promoting the formation of more ordered structures typical of cellulose type II. According to Mengesha (2022), this type of cellulose is associated with the reprecipitation of cellulose after alkaline hydrolysis [14]. This behavior can be explained by the ability of NaOH to break intermolecular and intramolecular bonds in cellulose, facilitating the reorganization of polymer chains into a new crystalline structure.

Another point is that the intensity of the peak at 2θ = 22.5° is greater in samples treated with NaOH compared to those treated with H_2_O_2_, indicating a higher degree of crystallinity in the former. This difference in peak intensity may be attributed to the greater efficiency of NaOH in removing impurities and amorphous components, resulting in a well-defined crystalline structure. According to Xie et al. (2016), the cellulose obtained has a defined crystalline structure, which aligns with the increased crystallinity index compared to the initial GBD sample, with alkaline chemical treatment being initially suitable for obtaining cellulose with a crystallinity comparable to results previously reported by other researchers [25]. In contrast, the treatment with H_2_O_2_, being a milder oxidizing agent, may not completely remove these impurities, leaving a higher proportion of amorphous regions and thus reducing the crystallinity index.

On the other hand, the chemical extraction with 8% H_2_O_2_ (sample CE-H08) shows a significant increase in the crystallinity index, reaching 42.2%. This increase can be attributed to the action of H_2_O_2_, which helps to remove lignin and other impurities, thereby improving the structural organization of the cellulose. An even greater increase in the crystallinity index is observed with chemical extraction using 25% and 30% NaOH (samples CE-Na25 and CE-Na30), which present crystallinity indices of 57.0% and 67.9%, respectively. NaOH is more effective in breaking bonds and removing amorphous components such as hemicellulose and lignin, resulting in a more crystalline structure. This is consistent with other studies where it is supported that these polymers are effectively dissolved through alkaline treatments, thereby increasing the crystallinity of the final material [13,39]. These values are comparable to and, in the case of CE-Na30, even higher than the crystallinity index of commercial eucalyptus fiber (ESCF), which is 51.5%, demonstrating that this particular chemical treatment is very effective at removing non-cellulosic components, being comparable to what has been previously reported in various investigations.

The application of conventional ultrasound during the chemical extraction with 8% H_2_O_2_ (CU-H08) significantly improves the crystallinity index to 66.7%, which is comparable to the commercial pine fiber (PLCF) with a crystallinity index of 66.4%. This increase can be attributed to ultrasonic cavitation, which enhances reagent penetration and removal of amorphous components [12,31]. In contrast, the sample CU-Na30, where 30% NaOH was used with conventional ultrasound, shows a surprisingly low crystallinity index of 20.2%. This anomalous result could be due to excessive degradation of cellulose under extremely harsh conditions, leading to the destruction of crystalline regions. On the other hand, the use of focused ultrasound results in the highest crystallinity indices among all samples. The sample FU-H08 has a crystallinity index of 76.6%, and the FU-Na25 sample reaches the highest value of 79.6%. These methods combine chemical action with ultrasonic energy, which promotes more efficient removal of amorphous components and optimal restructuring of cellulose chains. This is consistent with reports by Zope G. et al. [13] and Xie J. et al. [22], which demonstrate that ultrasonic treatments, in addition to allowing the removal of amorphous components, do not alter the crystalline structure of cellulose, which may be related to the applied power during treatment [13,25]. However, increasing the NaOH concentration to 30% (sample FU-Na30) results in a decrease in the crystallinity index to 37.0%, possibly due to the same over-degradation observed with conventional ultrasound.

Therefore, chemical extraction methods assisted by focused ultrasound, particularly with 25% NaOH (FU-Na25), result in the highest crystallinity indices, even surpassing those of commercial pine and eucalyptus cellulose fibers. The combination of chemical treatment and focused ultrasound proves to be the most effective for improving the crystallinity of cellulose extracted from banana pseudostem. On the other hand, chemical extraction assisted by conventional ultrasound and high concentrations of NaOH shows notable variability, suggesting that treatment conditions must be carefully optimized to avoid excessive degradation of the crystalline component.

### 3.3. Scanning Electron Microscopy (SEM)

**Figure 2:**
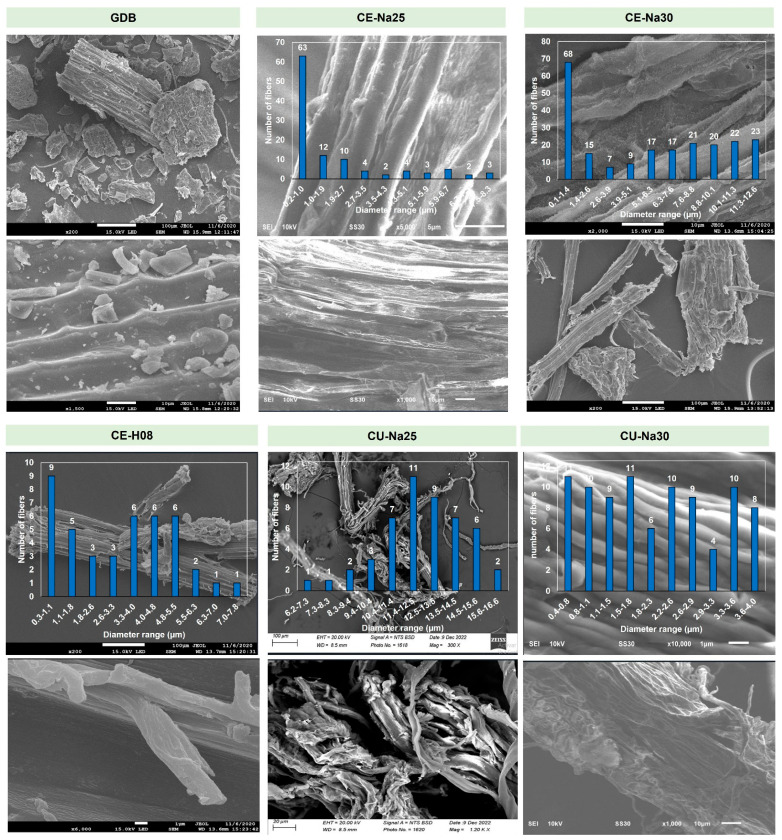

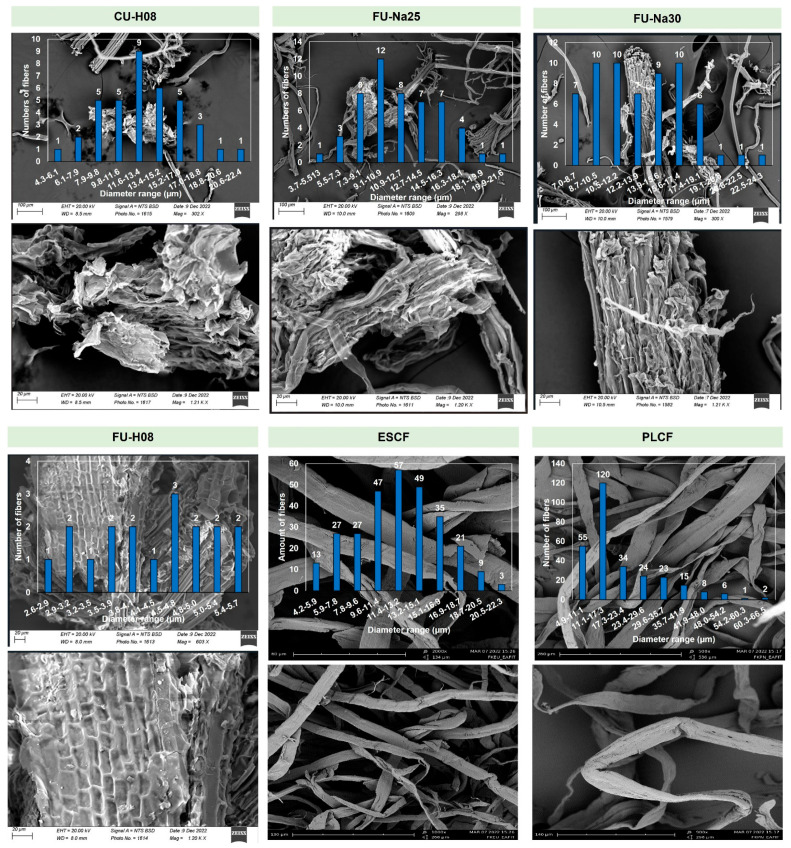
SEM images of the untreated biomass and the samples obtained with the different methods applied.

The micrographs obtained using scanning electron microscopy (SEM) revealed significant changes in the surface morphology of the cell wall in banana pseudostem samples, both untreated and treated with H_2_O_2_ and NaOH, using the different methods employed in this study (Figure 2). The removal of high fractions of lignin and hemicellulose following traditional and ultrasound-assisted treatments, both conventional and focused, induced structural alterations compared to the biomass extracted from the pseudostem. Previous studies, such as that by Thokchom et al., have demonstrated the extraction of cellulose fibers from banana flowers using ultrasound-assisted chemical methods [10].

In the untreated biomass (GDB), a compact structure with dense and packed components is observed, attributable to the cohesion of hemicellulose and lignin. Lignin is distinguished on the surface, resulting in a rough surface and somewhat altered fibrous organization, possibly due to the presence of other materials in the form of small irregular granules of varying sizes. Additionally, the surface exhibits some polarity and hydrophilic nature, which is consistent with the analysis of the extractable chemical composition.

In the biomass treated with 25% NaOH (CE-Na25), a slight morphological change on the surface is observed, related to the effect of the alkaline treatment that enhances cellulose accessibility, making delignification processes less aggressive and preserving the cellulose structure. Free cellulose fibers are not observed; instead, agglomerations are evident, possibly due to the residual presence of hemicellulose. The alkaline treatment yielded fibers with diameters predominantly between 0.2 and 1.0 µm, with a maximum diameter ranging from 7.5 to 8.3 µm.

The sample treated with 30% NaOH (CE-Na30) exhibits a slightly more defined surface than CE-Na25 and a larger fiber diameter. Agglomerations and clusters of cellulose are observed, but there are also some free fibers with a smoother appearance, associated with complete delignification, possibly due to the increased NaOH concentration, which enhances accessibility to the polymeric matrix and breaks chemical bonds between cellulose, hemicellulose, and lignin. According to Pragasam et al., these alkaline treatments facilitate the isolation of cellulose from plant polymeric matrices, increasing the surface exposure of the fibers [5,17]. The average diameter of the fibers in CE-Na30 showed a slight increase, with minimum values between 0.1 and 1.4 µm and maximum values between 11.3 and 12.6 µm.

For the sample treated with H_2_O_2_ (CE-H08), disorganization on the surface of the material is observed compared to the untreated biomass. This less aggressive treatment partially removes hemicellulose and lignin, resulting in a superficial fragmentation of the material with the presence of clusters and no free or smooth fibers. The fiber diameter in CE-H08 does not differ significantly from CE-Na25 but shows a slight difference compared to CE-Na30, with minimum diameters between 0.3 and 1.1 µm and maximum diameters between 7.0 and 7.8 µm.

Among conventional chemical treatments, the use of 30% NaOH (CE-Na30) proved to be the most effective for the removal of hemicellulose and the exposure of cellulose fibers, while hydrogen peroxide treatment (CE-H08) resulted in a significant alteration of the surface. The untreated biomass (GDB) showed a compact and dense structure, with a rough and fibrous surface. NaOH treatments improved accessibility and altered the surface morphology, with CE-Na30 being the treatment with the most pronounced effect.

In the samples treated with conventional sonication, the Cu-Na25 sample exhibited greater fiber release and surface exposure, although with less compact clusters. Conventional sonication facilitated the breaking of interactions between cellulose, lignin, and hemicellulose, resulting in a more defined surface with somewhat cross-linked fibers that were not very smooth. The fiber diameter in Cu-Na25 showed a considerable increase, with minimum values between 6.2 and 7.3 µm and maximum values between 15.6 and 16.6 µm, with most fibers between 11.4 and 12.5 µm.

The Cu-Na30 sample exhibited a more defined fibrillar formation and a clearer surface, with intertwinings and smoother fibers, although not completely isolated. The total removal of lignin and hemicellulose allowed for a slight decrease in the average fiber diameter, with minimum values between 0.4 and 0.8 µm and maximum values between 3.6 and 4.0 µm, with most fibers having diameters between 1.5 and 1.8 µm. According to Jyoti et al., bleaching and ultrasound treatment facilitates the removal of amorphous compounds such as lignin and hemicellulose, contributing to greater surface exposure of cellulose [10].

For the CU-H08 sample, the obtained material showed superficial agglomerations attributed to traces of hemicellulose, clusters, and a lack of clear fiber isolation, in contrast to CE-H08 and CU-H08, which exhibited lower hemicellulose content. The fiber diameter distribution was broader, with minimum values between 4.3 and 6.1 µm and maximum values between 20.6 and 22.4 µm, with most fibers between 11.6 and 13.4 µm, indicating an increase in average diameter compared to previous samples.

In the samples treated with focused sonication, the FU-Na25 sample displayed irregular morphology and compact cellulosic material in clumps, with no smooth or isolated fibers, attributed to a high percentage of hemicellulose that was not removed due to the limited accessibility of NaOH in the polymeric matrix of the pseudostem. The fiber diameter distribution was broad, with minimum values between 3.7 and 5.5 µm and maximum values between 19.9 and 21.6 µm.

The FU-Na30 sample exhibited a morphology with somewhat compact cellulosic material and some clumps, but with the release of a few fibers with a smoother surface appearance. The increase in NaOH concentration and the cavitation phenomenon facilitated the removal of lignin and exposure of cellulose, although small traces of hemicellulose persisted. This resulted in a considerable increase in fiber diameter, with minimum values between 7.0 and 8.7 µm and maximum values between 22.5 and 24.3 µm, with most fibers ranging from 8.7 to 17.4 µm. This result is comparable to that of Yashim et al., who established that the combination of chemical treatments with focused ultrasound induces changes in the fibrous structure of cellulose [36]. Additionally, studies by Yang et al. suggest that alkaline treatment assisted with focused sonication may induce spiral hydrolysis on cell wall surfaces, promoting cell wall separation and the formation of a rod-like isolated system, consistent with the findings for fibers in FU-Na25 and FU-Na30 [27].

Finally, the FU-H08 sample showed that focused sonication was not decisive for the complete removal of hemicellulose, demonstrating cellulosic material agglomerations and a lack of clear release of smooth fibers. The fiber diameter distribution was lower and more uniform, with average values between 4.5 and 4.8 µm and maximum values between 5.4 and 5.7 µm, suggesting that focused sonication is not as effective as treatments CE-H08 and CU-H08 for complete hemicellulose removal.

In conclusion, conventional chemical treatments and treatments assisted by conventional and focused sonication significantly influenced the morphology, surface exposure, and diameters of cellulose fibers extracted from banana pseudostem. Conventional sonication combined with alkaline treatments improved the definition and exposure of fibers, while focused sonication allowed for obtaining more uniformly sized fibers, though it presented challenges in the complete removal of hemicellulose. These results suggest that the choice of treatment method and specific conditions are crucial for optimizing the properties of cellulose fibers for applications in the paper industry.

### 3.4. Thermogravimetric Analysis (TGA)

**Figure 3:**
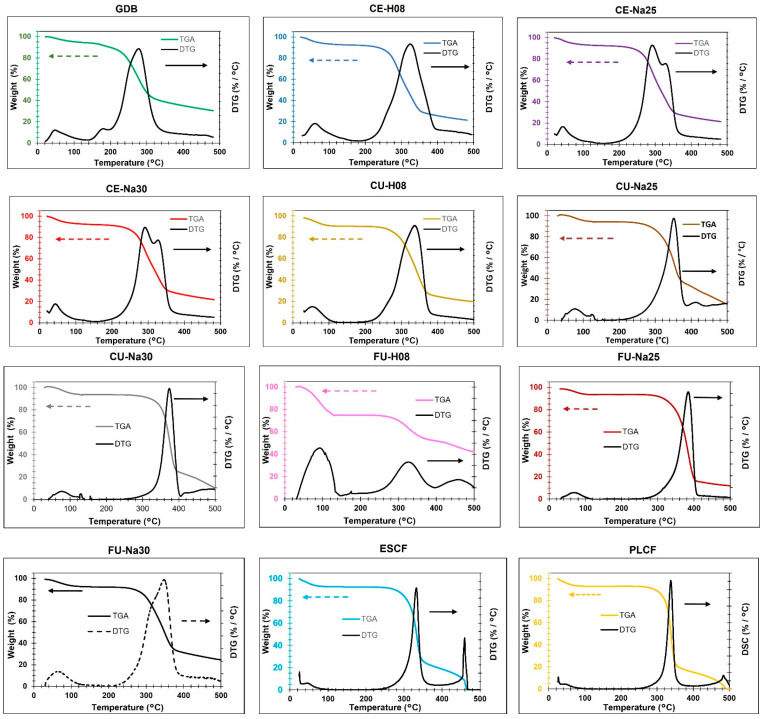
Thermogravimetric curves of the untreated biomass and the samples obtained with the different methods applied.

Properties are important for various applications, especially if the cellulose is intended for use as a reinforcement material. Additionally, weight loss events reflect the degradation of different cellulose components. Based on the thermograms obtained for each sample (Figure 3), three weight loss events were identified along with their respective percentages and temperature ranges (Table 3). The temperature range for the first weight loss event is between 25 and 125 °C and is associated with the loss of free and physically bound water in the cellulose [21]. The removal of moisture is an endothermic process, meaning that there is no significant chemical decomposition in this range. In similar studies, the initial weight loss is attributed to the removal of adsorbed moisture and interstitial water [21]. For example, Prerna et al. [25] report that the loss of adsorbed water occurs in the range of 25–150 °C.

The temperature range for the second weight loss event is between 215 and 415 °C and is associated with the decomposition of hemicellulose and cellulose [27]. Hemicellulose begins to decompose around 200–260 °C, while cellulose decomposes in the range of 260–360 °C. According to Merais et al. [43], hemicellulose decomposes in the range of 200–260 °C, while cellulose shows significant weight loss between 260 and 360 °C due to the breakdown of its glucose chains. Our samples reveal significant weight loss (20–78%) in this interval, reflecting the decomposition of hemicellulose and cellulose. For example, the FU-Na25 sample shows a 78% loss in this range, indicating a high concentration of cellulose undergoing thermal decomposition. The third weight loss event occurs between 330 and 500 °C and can be primarily associated with the decomposition of lignin and the carbonization of carbonaceous residues. Lignin decomposes over a broader temperature range (200–500 °C), but most of its decomposition occurs at temperatures above 300 °C. Lignin undergoes slow and continuous thermal decomposition from 200 °C to 500 °C, as reported by Lu Q. et al. [30].

For the GDB sample, weight loss events occurred in the intervals of 25–215 °C (11%), 215–330 °C (49%), and 330–500 °C (10%). This behavior suggests that cellulose in its natural state has limited thermal stability, with initial decomposition at lower temperatures, likely due to the presence of non-cellulosic and amorphous components in the untreated material. This is consistent with findings by Basumatary et al., which report that the greatest weight loss occurs at temperatures around 330 °C for such materials, associated with their complete degradation. This aligns with the type and nature of the treated sample and corresponds with the results obtained in this study [16].

For the CE-H08 sample, weight loss events were recorded in the intervals of 50–120 °C (5%), 250–400 °C (59%), and 400–500 °C (7.5%). The increase in crystallinity and decomposition temperature suggests that the treatment with H_2_O_2_ improves thermal stability by removing amorphous components and enhancing the structural organization of cellulose. The CE-Na25 and CE-Na30 samples, extracted with 25% and 30% NaOH, respectively, exhibited crystallinity indices of 57.0% and 67.9%. Weight loss events for CE-Na25 occurred at 45–110 °C (7%), 220–360 °C (63%), and 360–500 °C (7%), while for CE-Na30 they were at 45–100 °C (7%), 220–370 °C (62%), and 370–500 °C (6%). The thermal degradation profiles observed in chemically treated samples are comparable to those reported by Elanthikkal [21] and Mengesta [39], who also showed significant cellulose component degradation around 400 °C due to depolymerization, dehydration, and decomposition of glycosidic units. Furthermore, comparing with previous research, the residual decomposition shows a slow degradation profile with final residual values that are very similar. Overall, the thermal degradation profiles for samples treated in alkaline media exhibit similar patterns [14,33]. These results indicate greater thermal stability compared to the untreated sample and the one treated with H_2_O_2_, likely due to the increased removal of hemicellulose and lignin, resulting in a more crystalline and heat-resistant structure [18].

For the samples treated with conventional ultrasound (CU-H08, CU-Na25, and CU-Na30), the crystallinity indices were 66.7%, 57.5%, and 20.2%, respectively. CU-H08, with 8% H_2_O_2_, showed weight loss events at 50–100 °C (9%), 240–385 °C (64%), and 385–500 °C (6%). CU-Na25 and CU-Na30, treated with NaOH, exhibited events at 60–125 °C (6%), 280–380 °C (59%), and 380–500 °C (21%), and 50–130 °C (6%), 310–405 °C (68%), and 405–500 °C (14%), respectively. The application of conventional ultrasound appears to increase the efficiency of chemical treatment, particularly in terms of crystallinity and thermal stability, especially for the sample treated in alkaline media with 25% NaOH, which is also comparable in terms of crystallinity according to Thokchom et al. [10], associated with the conditions employed in ultrasound. However, the CU-Na30 sample shows an anomaly with lower crystallinity, which might be related to a degrading effect of ultrasound at high NaOH concentrations. According to previous research by Chen et al. [27], thermal degradations in these treatments depend on the conditions under which they are performed and the starting material, making it consistent that the CU-Na30 sample showed a decrease in crystallinity, although its thermal degradation pattern is similar to the other samples [10,27].

The FU-H08, FU-Na25, and FU-Na30 samples treated with focused ultrasound had crystallinity indices of 76.6%, 79.6%, and 37.0%, respectively. The weight loss events for FU-H08 occurred at 50–125 °C (25%), 275–370 °C (20%), and 370–500 °C (12%), while for FU-Na25 and FU-Na30 they were at 55–110 °C (6%), 275–415 °C (78%), and 415–500 °C (4%), and 55–125 °C (8%), 245–385 °C (59%), and 385–500 °C (7%), respectively. Treatments assisted by focused ultrasound provide higher crystallinity and thermal stability, particularly in the FU-Na25 sample, where high crystallinity is correlated with greater resistance to thermal degradation.

Commercial eucalyptus (ESCF) and pine (PLCF) samples had crystallinity indices of 51.5% and 66.4%, respectively. Weight loss events for ESCF occurred at 30–90 °C (7%), 270–365 °C (68%), and 365–470 °C (23%), while for PLCF they were at 35–80 °C (7%), 285–370 °C (73%), and 370–490 °C (18%). Commercial fibers show thermal stability comparable to samples treated with focused ultrasound, suggesting that advanced extraction methods can produce cellulose with thermal properties similar to commercial grades.

Chemical treatments with H_2_O_2_ and NaOH show a clear effect on the crystallinity and thermal stability of the extracted cellulose. NaOH treatments resulted in higher crystallinity indices compared to H_2_O_2_. For example, the CE-Na30 sample exhibited a crystallinity index of 67.9%, significantly higher than the 42.2% of the CE-H08 sample treated with H_2_O_2_. This increase in crystallinity is due to the more effective removal of lignin and hemicellulose by NaOH, which promotes the reorganization of cellulose chains into a more crystalline and ordered structure. This crystallinity index is similar to that reported by Chen et al., who found crystallinity indices between 60 and 70% for fibers treated in alkaline medium [27]. The higher crystallinity, in turn, translates into greater thermal stability, as the crystalline regions of cellulose are more resistant to thermal decomposition. This is comparable to the results found in the research conducted by Flores et al., where chemical treatments on biomass material show crystalline patterns with more defined peaks, suggesting a higher degree of crystallinity. This is consistent with a greater amount of cellulose obtained, corroborating the removal of hemicellulose and lignin, primarily in samples treated in alkaline medium [23]. In NaOH-treated samples, the second weight loss event, corresponding to the main decomposition of cellulose, occurs at higher temperatures and with greater percentages of weight loss (e.g., CE-Na25: 220–360 °C, 63%). This indicates greater thermal resistance and a more complete decomposition of purified cellulose.

The use of conventional ultrasound in chemical extraction improves crystallinity and thermal stability compared to methods without ultrasound. For example, CU-H08 (66.7%) showed higher crystallinity and thermal stability than CE-H08 (42.2%). Ultrasound helps to break down microfibrils and facilitates the penetration of the chemical agent, resulting in more efficient removal of amorphous components and a greater reorganization of cellulose chains. This supports previous descriptions and demonstrates that energy-assisted treatment somehow favors a better crystalline conformation of the final material, as supported by prior research conducted by Yashim et al. [36]. Focused ultrasound produces even higher crystallinity and thermal stability; however, it is important to highlight that for the CU-Na30 sample, crystallinity decreased slightly compared to other samples treated in an alkaline medium and assisted by ultrasound. This is consistent with findings by Freitas et al. [24], possibly due to the NaOH concentration promoting the swelling of interfibrillar regions, making them somewhat less rigid. This phenomenon, in turn, allows for the reorganization of cellulose due to the penetration of Na ions into the crystalline network, forming sodium cellulose antiparallel crystalline complexes that alter the crystallinity of the material. The FU-Na25 sample, with a crystallinity index of 79.6%, shows superior thermal resistance, with its second weight loss event at temperatures of 275–415 °C and a high weight loss percentage (78%). This is due to the ability of focused ultrasound to generate intense and focused cavitation, enhancing chemical interaction and promoting greater purification and crystallization of cellulose. This is consistent with what Liu et al. [38] reported, where, in addition to the discussed findings, the high crystallinity value for the FU-Na25 sample suggests that ultrasound facilitates the solubilization of hemicellulose in the alkaline solution, resulting in purer and more crystalline cellulose.

There is a direct correlation between the crystallinity index and the thermal stability of cellulose samples. Higher crystallinity is associated with greater resistance to thermal decomposition, and weight loss events occur at higher temperatures. This is due to the more ordered and compact structure of crystalline regions, which require more energy to decompose. Commercial eucalyptus (ESCF) and pine (PLCF) fibers exhibited crystallinity indices of 51.5% and 66.4%, respectively, and demonstrated thermal stability comparable to samples treated with focused ultrasound. This suggests that advanced extraction methods, such as focused ultrasound, can produce cellulose with thermal properties similar to those of commercial fibers, indicating a positive potential for the commercialization of cellulose obtained from agricultural residues.

In other words, chemical extraction methods, especially when assisted by ultrasound (conventional and focused), significantly enhance thermal stability and crystallinity of cellulose extracted from banana pseudostem residues. Treatments with 25% and 30% NaOH, assisted by focused ultrasound, yield the best results in terms of high crystallinity and thermal resistance. These findings suggest that focused ultrasound is a promising technique for producing high-quality cellulose from agricultural residues, comparing favorably with commercially available fibers. The comparative analysis of weight loss data across different temperature ranges reveals that extraction and treatment methods significantly influence the composition and thermal stability of cellulose. Ultrasound-assisted chemical treatments, especially focused ultrasound, result in cellulose with higher crystallinity and thermal stability. The correlation between extraction methods, crystallinity index, and thermal stability provides a solid foundation for optimizing extraction processes and enhancing material properties for industrial applications.

## 4. Conclusions

Treatment with NaOH, particularly at 30%, resulted in the highest cellulose yields, reaching up to 99.5%. In contrast, treatments using H_2_O_2_ achieved lower cellulose percentages, ranging from 60% to 68%. The combination of NaOH with conventional sonication proved to be the most effective for maximizing pure cellulose recovery. Conventional sonication demonstrated greater efficacy in achieving high cellulose percentages, whereas focused sonication tended to reduce cellulose content, likely due to partial fiber degradation under extreme conditions. The evaluated techniques, particularly ultrasound-assisted methods with NaOH, achieved yields and purities of cellulose comparable to or exceeding those of commercial pine and eucalyptus fibers. This highlights the potential of these methods for industrial applications. The combination of chemical treatments with conventional ultrasound emerges as the optimal strategy for obtaining high-purity and high-yield cellulose. Although focused sonication is useful, it did not show significant advantages over conventional sonication and may lead to cellulose degradation. For achieving high-purity and high-yield cellulose, the use of 30% NaOH combined with conventional sonication is recommended. Optimization of treatment parameters is crucial to prevent cellulose degradation and maximize process efficiency.

## Figures and Tables

**Table 1:** Codes for the samples obtained according to the different evaluated procedures.

Code	Extraction Process
GDB	Ground dry biomass powder
CE-Na25	Conventional extraction with 25% NaOH
CE-Na30	Conventional extraction with 30% NaOH
CE-H08	Conventional extraction with 8% H_2_O_2_
CU-Na25	Extraction assisted with conventional ultrasound and 25% NaOH
CU-Na30	Extraction assisted with conventional ultrasound and 30% NaOH
CU-H08	Extraction assisted with conventional ultrasound and H_2_O_2_ 8%
FU-Na25	Extraction assisted with focused ultrasound and 25% NaOH
FU-Na30	Extraction assisted with focused ultrasound and 30% NaOH
FU-H08	Extraction assisted with focused ultrasound and H_2_O_2_ 8%
ESCF	Eucalyptus short commercial fiber
PLCF	Pine long commercial fiber

**Table 2:** Chemical composition of cellulose samples after each chemical treatment.

Code	Yield (%)	Humidity (%)	Extractives (%)		Chemical Composition (%)				Cellulose Global Yield (%)	Crystallinity Index (%)
			Water	Ethanol	Lignin	Holocellulose	Hemicellulose	Cellulose		
GDB	29.5	15.82	33.49	47.85	30.06	59.22	18.16	41.06	-	8.2
CE-Na25	13.3	9.97	1.92	1.39	0	92.91	4.24	88.68	11.79	42.4
CE-Na30	12.0	8.89	1.65	0.16	0	93.59	3.27	90.32	11.18	57.0
CE-H08	45.2	8.33	13.53	3.47	0	67.97	7.47	60.50	27.35	67.9
CU-Na25	16.3	5.36	1.97	2.08	0	85.75	0.0	89.52	14.59	66.7
CU-Na30	18.5	9.69	3.17	1.15	0	59.49	0.0	99.50	18.42	57.5
CU-H08	49.3	9.24	3.76	0.56	0	70.34	2.22	68.12	33.58	20.2
FU-Na25	18.3	11.11	1.36	1.95	0	89.39	14.14	75.25	13.77	76.6
FU-Na30	20.0	8.65	2.07	0.04	0	82.45	2.29	80.16	16.03	79.6
FU-H08	46.6	9.34	2.57	1.61	0	73.77	7.97	65.80	30.66	37.0
ESCF	-	10.22	2.04	7.21	0	84.91	7.39	77.52	-	51.5
PLCF	-	8.67	2.58	4.94	0	86.57	9.42	77.15	-	66.4

**Table 3:** Thermal decomposition events detected for the different cellulosic materials.

Code	Detected Weight Loss Thermal Events									Residue Weight (%)
	First Event			Second Event			Third Event			
	Temperature (°C)		Weight Loss (%)	Temperature (°C)		Weight Loss (%)	Temperature (°C)		Weight Loss (%)	
	Range	Maximum		Range	Maximum		Range	Maximum		
GDB	45–215	45.9	11.0	215–330	274.4	49.5	330–500	462.8	10.2	30.6
CE-H08	50–120	64.9	5.0	250–400	325.8	59.3	400–500	425.4	7.5	26.7
CE-Na25	45–110	40.5	7.0	220–360	290.1	63.2	360–500	411.9	7.3	21.7
CE-Na30	45–100	43.9	7.1	220–370	293.8	62.1	370–500	402.3	6.0	21.8
CU-H08	50–100	48.3	9.2	240–385	393.4	64.0	385–500	448.9	6.3	19.7
CU-Na25	60–135	73.9	6.2	280–380	354.4	59.3	380–500	408.9	21.2	18.8
CU-Na30	50–130	85.6	6.1	310–405	376.7	68.3	405–500	491.2	14.3	12.4
FU-H08	50–125	98.7	25.0	275–370	337.6	20.1	370–500	462.8	12.4	42.4
FU-Na25	55–110	80.8	6.0	275–415	385.8	78.4	415–500	-	4.1	5.1
FU-Na30	55–125	67.9	8.3	245–385	345.3	59.3	385–500	461.9	7.4	24.7
ESCF	30–90	47.2	7.2	270–365	332.5	68.2	365–470	461.3	23.1	0
PLCF	35–80	53.1	7.1	285–370	338.9	73.4	370–490	486.4	18.1	0

## Data Availability

Data are contained within the article.

## Supplementary Materials

The following supporting information can be downloaded at: https://www.mdpi.com/article/10.3390/polym16192785/s1, Figure S1. ATR-FTIR spectra of GDB, Figure S2. ATR-FTIR spectra of CE-H08, Figure S3. ATR-FTIR spectra of CE-Na25, Figure S4. ATR-FTIR spectra of CE-Na30, Figure S5. ATR-FTIR spectra of CU-H08, Figure S6. ATR-FTIR spectra of CU-Na25, Figure S7. ATR-FTIR spectra of CU-Na30, Figure S8. ATR-FTIR spectra of FU-H08, Figure S9. ATR-FTIR spectra of FU-Na25, Figure S10. ATR-FTIR spectra of FU-Na30, Figure S11. ATR-FTIR spectra of ESCF, Figure S12. ATR-FTIR spectra of PLCF.
