# Supplementary material for: Enhanced Cellulose Extraction from Banana Pseudostem Waste: A Comparative Analysis Using Chemical Methods Assisted by Conventional and Focused Ultrasound
Source: Polymers (Basel). 2024 Sep 30;16(19):2785. doi: 10.3390/polym16192785 (PMC11479204; doi:10.3390/polym16192785)
Supplement: Supplementary file 1 [file polymers-16-02785-s001.zip › polymers-3226429-supplementary.pdf]

polymers MDPI

Enhanced Cellulose Extraction from Banana Pseudostem Waste: A Comparative Analysis Using Chemical Methods Assisted by Conventional and Focused Ultrasound

SUPPLEMENTARY INFORMATION

1. Methodology (Materials and Methods)

1.1 Attenuated Total Reflectance Fourier Transform Infrared Spectroscopy (ATR-FTIR) Analysis

ATR-FTIR analysis was used to determine the chemical bonds, chemical nature, and functional groups of extracted cellulose. IR Shimadzu IRAffinity-1S spectrometer was used to perform the analysis. The spectral analysis was carried out between the wavenumber range of 800–4000 cm^{-1} at a scanning rate of 50 cm^{-1}/s with a spectral resolution of 8 cm^{-1}.

2. Results and discussions

2.1. Attenuated Total Reflectance Fourier Transform Infrared Spectroscopy (ATR-FTIR) Analysis

Figures S1a-S1l illustrate the FTIR-ATR spectra of the different samples, where the presence or absence of the following bands with their respective assignments can be identified. This allows for the inference of the preservation of the basic chemical structure of cellulose even after chemical treatments, as well as the evaluation of the efficiency in removing lignin, hemicellulose, and other components during the cellulose extraction process: 2870 cm^{-1}: C-H stretching in CH_3 and CH_2, 1732 cm^{-1}: C=O stretching in esters, aldehydes, ketones (hemicellulose/lignin), 1640 cm^{-1}: C=O stretching in amides or absorbed water in cellulose, 1600 cm^{-1}: aromatic C=C stretching (lignin), 1515 cm^{-1}: aromatic C=C stretching (lignin), 1465 cm^{-1}: C-H bending in CH_3 and CH_2, and possibly bending in aromatic rings of lignin, 1365 cm^{-1}: C-H bending in CH_3 and CH_2, deformation of cellulose/hemicellulose, 1170 cm^{-1}: attributed to asymmetric C-O-C stretching vibrations associated with type I cellulose, 1050 cm^{-1}: C-O stretching in alcohols (cellulose/hemicellulose), 990 cm^{-1}: C-H out-of-plane bending vibrations, 950 cm^{-1}: torsional vibrations of β-glucosidic C-O-C bonds in the cellulose ring structure, 900 cm^{-1}: vibrations associated with β-(1→4)-glucan units in cellulose [1] [2].

The GDB sample reveals a high intensity in the band at 1600 cm^{-1}, associated with aromatic C=C stretching of lignin, indicating a high presence of lignin. The absence of bands at 2870 cm^{-1} (C-H in CH_3 and CH_2), 1732 cm^{-1} (C=O in hemicellulose/lignin), 1640 cm^{-1} (C=O in amides/absorbed water), 1515 cm^{-1} (aromatic C=C of lignin), and 1050 cm^{-1} (C-O in cellulose/hemicellulose) suggests an unmodified lignocellulosic structure. The presence of the band at 1365 cm^{-1} indicates C-H bending in CH_3 and CH_2, and some deformation of cellulose/hemicellulose, though in smaller amounts compared to treated samples, while the high intensity of the band at 1050 cm^{-1} suggests the presence of alcohols in cellulose and hemicellulose. This confirms that GDB is a raw material with a high proportion of lignin and a lower amount of accessible cellulosic components.

The CE-H08 sample presents a band at 2870 cm^{-1}, although with lower intensity than ESCF and PLCF, indicating a reduction in CH_3 and CH_2 groups. The band at 1732 cm^{-1}

appears, suggesting residual hemicellulose/lignin presence. The band at 1640 cm^{-1}, related to water absorption, and the band at 1515 cm^{-1} (aromatic C=C of lignin) indicate partial removal of lignin. The high intensity of the band at 1365 cm^{-1} in the CE treatment samples indicates a higher cellulose content. The lower intensity of the band at 1150 cm^{-1} suggests a reduced amount of ether linkages in cellulose compared to ESCF and PLCF.

In the CE-Na25 sample, the band at 2870 cm^{-1} is less intense, and the bands at 1732 cm^{-1} and 1640 cm^{-1} are absent, indicating effective removal of hemicellulose and lignin. The band at 1600 cm^{-1} appears with low intensity, suggesting a reduction in aromatic lignin compounds. The band at 1365 cm^{-1} is more pronounced, indicating a higher presence of cellulose. The similarity in the intensity of the band at 1150 cm^{-1} with other chemically treated samples indicates consistency in the presence of ether linkages in cellulose.

The CE-Na30 sample shows low intensity at 2870 cm^{-1} and the absence of bands at 1732 cm^{-1}, 1640 cm^{-1}, and 1600 cm^{-1}, similar to CE-Na25, suggesting an almost complete removal of lignin and hemicellulose. The band at 1365 cm^{-1} is intense, indicating a high presence of cellulose. The band at 1150 cm^{-1} exhibits similar intensity to other NaOH-treated samples, confirming the presence of ether linkages in cellulose.

The CU-H08 sample does not present the band at 2870 cm^{-1}, and the band at 1732 cm^{-1} shows intensity similar to CU-Na25, indicating residual hemicellulose/lignin. The band at 1640 cm^{-1} appears with moderate intensity, while the band at 1515 cm^{-1} (aromatic C=C of lignin) indicates partial removal of lignin. The presence of the band at 1365 cm^{-1}, with lower intensity compared to CE treatments, suggests a lower amount of cellulose. The high intensity of the band at 1150 cm^{-1} also indicates a higher amount of cellulose compared to other treated samples.

The CU-Na25 sample does not show the band at 2870 cm^{-1}, and the band at 1732 cm^{-1} appears with lower intensity, indicating significant reduction of lignin and hemicellulose. The absence of the band at 1640 cm^{-1} and low intensity of the band at 1600 cm^{-1} suggest partial removal of lignin. The band at 1365 cm^{-1} is more pronounced compared to NaOH-treated samples without sonication, indicating a higher presence of cellulose. The similarity in intensity of the band at 1150 cm^{-1} with other chemically treated samples confirms the presence of ether linkages in cellulose.

In the CU-Na30 sample, significant bands for lignin (1600 cm^{-1} and 1515 cm^{-1}) are not identified, indicating effective removal of lignin. The presence of bands at 1365 cm^{-1} and 1150 cm^{-1} suggests a significant cellulose content. Additionally, the complete disappearance of the band at 1732 cm^{-1} confirms the total removal of hemicellulose, which is consistent with the chemical composition results. Furthermore, when comparing this band with those of commercial eucalyptus cellulose (ESCF) and commercial pine cellulose (PLCF), it is well-defined, suggesting that these commercial celluloses are not entirely pure and contain traces of hemicellulose, highlighting the effectiveness of the method employed in this study.

The FU-H08 sample does not present the band at 2870 cm^{-1}, and the band at 1732 cm^{-1} appears with lower intensity, indicating residues of lignin and hemicellulose, similar to the CU-H08 sample. The band at 1640 cm^{-1} has moderate intensity, indicating the presence of amides or absorbed water. The partial removal of lignin is reflected in the absence of the band at 1600 cm^{-1}. The presence of the band at 1365 cm^{-1}, with lower intensity compared to CE treatments, suggests a lower amount of cellulose. The high intensity of the band at 1150 cm^{-1} suggests the presence of ether linkages in cellulose.

The FU-Na25 sample does not show the band at 2870 cm^{-1}, and the band at 1732 cm^{-1} appears with lower intensity, indicating a reduction in lignin and hemicellulose. The absence of the band at 1640 cm^{-1} and the low intensity of the band at 1600 cm^{-1} suggest partial removal of lignin. The band at 1365 cm^{-1} is more pronounced compared to NaOH-treated samples without sonication, indicating a higher presence of cellulose. The similarity in the intensity of the band at 1150 cm^{-1} with other chemically treated samples confirms the presence of ether linkages in cellulose.

The FU-Na30 sample shows a band at 1640 cm^{-1} with low intensity, indicating effective removal of hemicellulose and lignin. The absence of significant bands for lignin (1600 cm^{-1} and 1515 cm^{-1}) and the band at 1465 cm^{-1} suggests effective removal of lignin. The presence of the band at 1365 cm^{-1}, though less intense than in CE samples, suggests a moderate cellulose content. The band at 1150 cm^{-1} indicates a moderate amount of accessible cellulose.

Commercial eucalyptus (ESCF) and pine (PLCF) fibers show bands at 2870 cm^{-1} (C-H in CH_3 and CH_2) with high intensity, indicating the presence of methyl and methylene groups. Bands at 1732 cm^{-1} suggest residual hemicellulose/lignin. The absence of bands at 1640 cm^{-1}, 1600 cm^{-1}, and 1515 cm^{-1} indicates effective removal of hemicellulose and lignin. The high intensity of bands at 1465 cm^{-1} and 1150 cm^{-1} suggests a higher proportion of cellulose, confirming that commercial fibers exhibit high cellulose purity with effective removal of lignin and hemicellulose, serving as references to evaluate the efficacy of the implemented extraction methods.

A general comparative analysis of the FTIR-ATR spectra reveals that chemical extraction methods, both ultrasonic-assisted and non-ultrasonic-assisted, exhibit varying degrees of efficacy in the removal of lignin and hemicellulose and in achieving high-purity cellulose. For instance, methods using 8% H_2O_2 (CE-H08 and CU-H08) demonstrate partial removal of lignin and hemicellulose, with higher cellulose purity observed in the ultrasonic-assisted CU-H08 sample. Methods employing 25% NaOH (CE-Na25 and CU-Na25) are effective in removing hemicellulose, with significant reduction of lignin, resulting in moderate to high cellulose content. Methods using 30% NaOH (CE-Na30 and CU-Na30) exhibit the most effective removal of lignin and hemicellulose, particularly in the CU-Na30 sample treated with ultrasonic-assisted conventional sonication, yielding high-purity cellulose. Focused ultrasonic-assisted methods (FU-H08 and FU-Na25) show partial removal of lignin and hemicellulose with results comparable to conventional methods but without a significant advantage in cellulose purity. In summary, chemical treatments assisted by ultrasonic conventional sonication, especially with 30% NaOH (FU-Na30), demonstrate greater efficacy in removing non-cellulosic components and obtaining high-purity cellulose.

In other words, the bands identified in the DRIFT spectra for each sample are consistent with the results obtained for chemical composition, demonstrating that both conventional and focused ultrasound show effective removal of lignin and hemicellulose, particularly with the 30% NaOH treatment. Furthermore, both methods exhibited significant cellulose content, with the band at 1365 cm^{-1} indicating a high cellulose content in the samples treated with 30% NaOH. In summary, both conventional and focused ultrasound methods are effective in removing lignin and hemicellulose and obtaining high-purity cellulose. However, there is no significant advantage of one method over the other in terms of cellulose purity. The choice between conventional and focused ultrasound may depend on other factors, such as energy efficiency, equipment cost, and industrial process preferences.

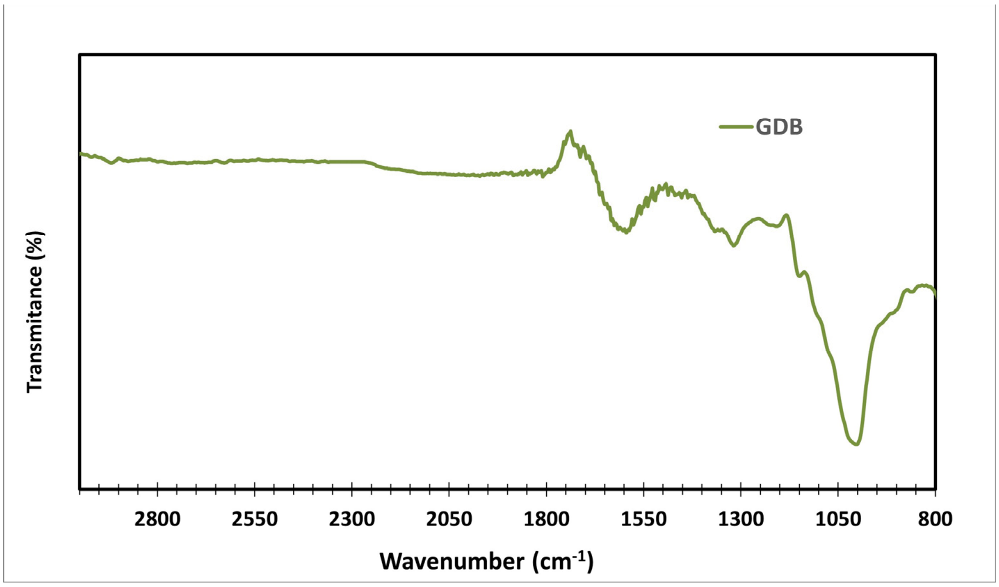

Figure S1. ATR-FTIR spectra of GDB.

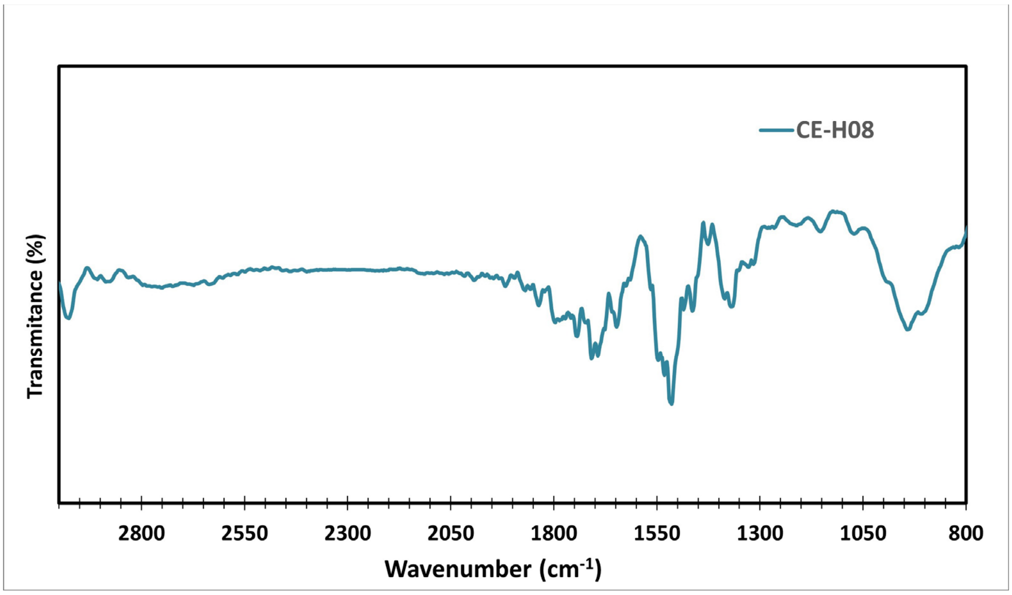

Figure S2. ATR-FTIR spectra of CE-H08.

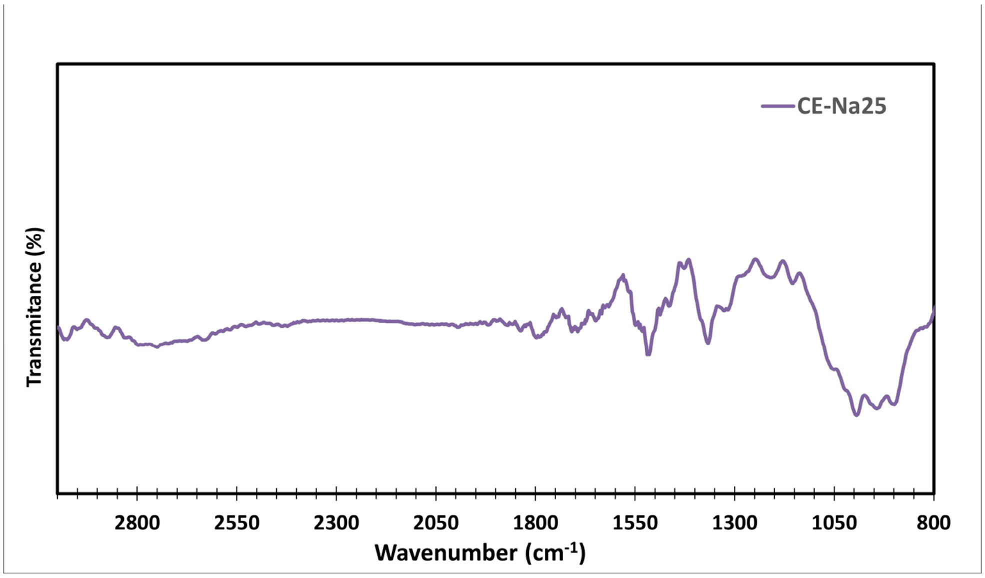

Figure S3. ATR-FTIR spectra of CE-Na25.

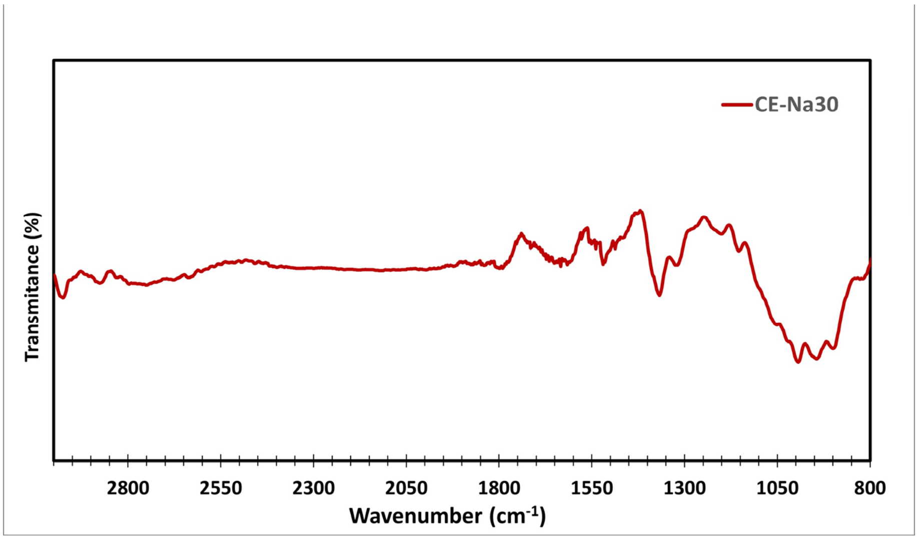

Figure S4. ATR-FTIR spectra of CE-Na30.

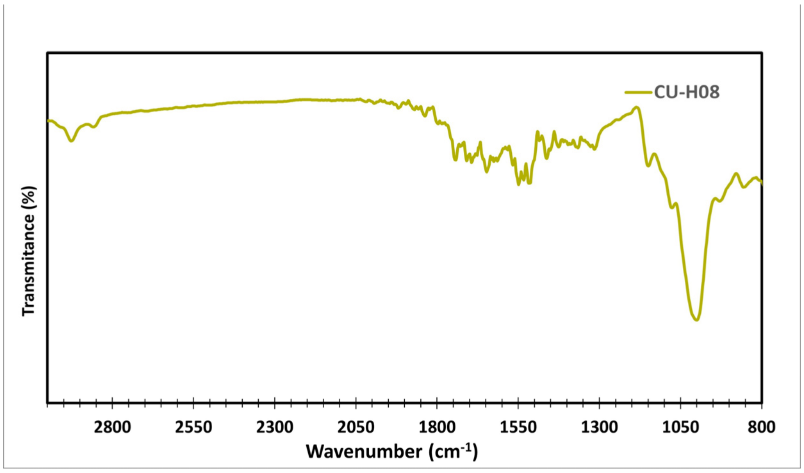

Figure S5. ATR-FTIR spectra of CU-H08.

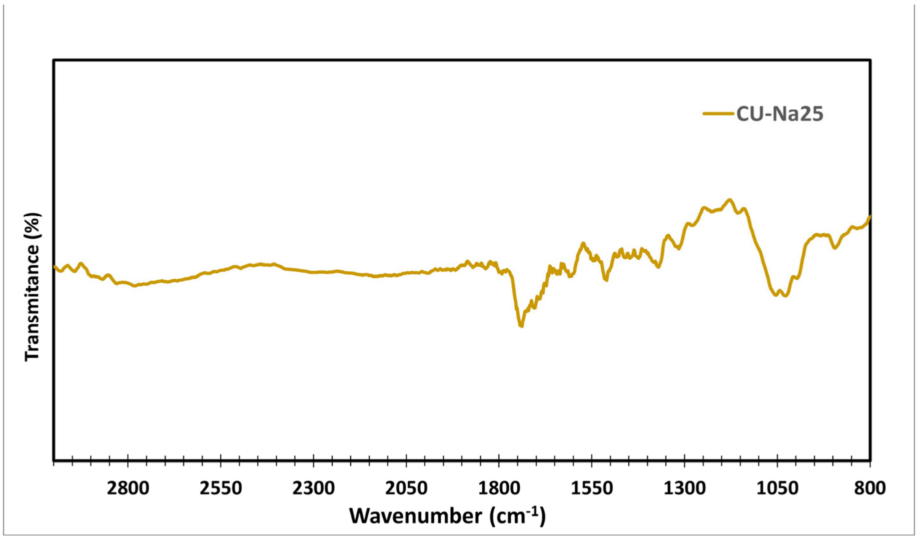

Figure S6. ATR-FTIR spectra of CU-Na25.

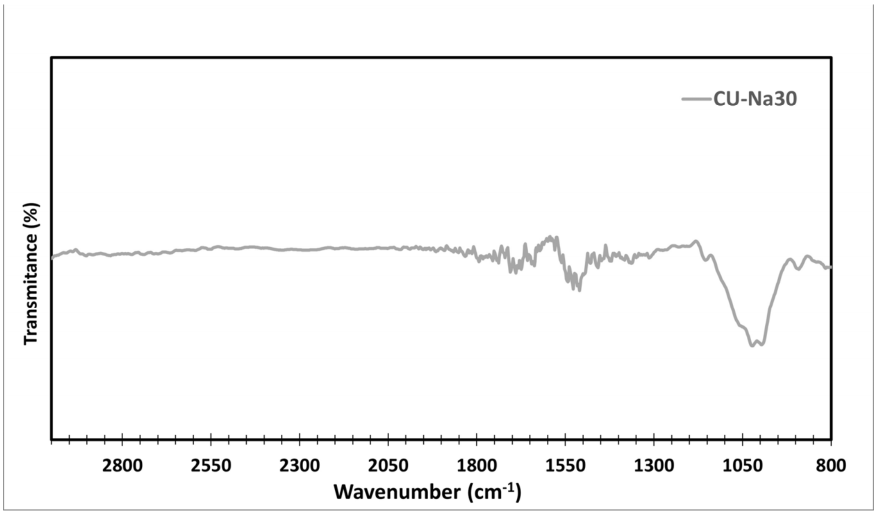

Figure S7. ATR-FTIR spectra of CU-Na30

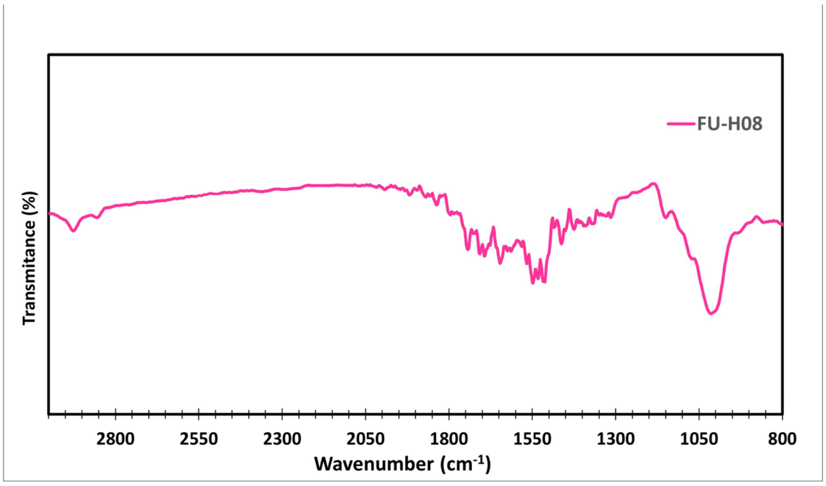

Figure S8. ATR-FTIR spectra of FU-H08.

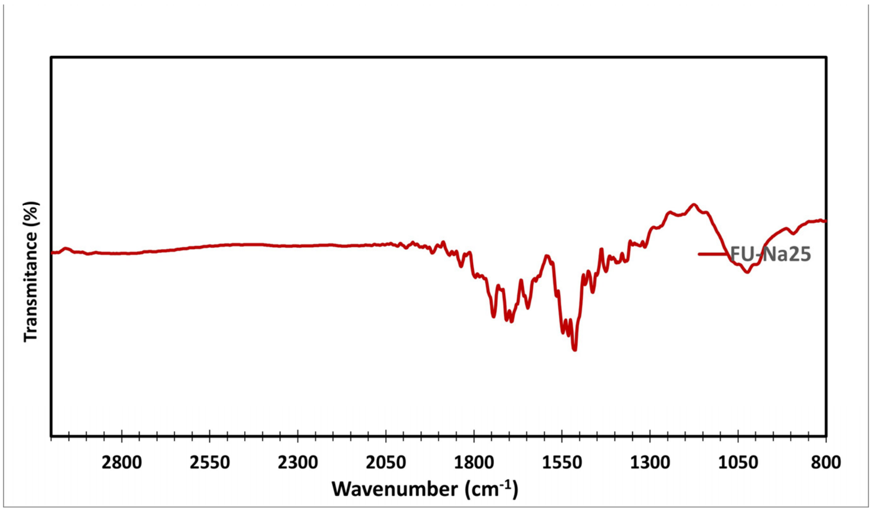

Figure S9. ATR-FTIR spectra of FU-Na25.

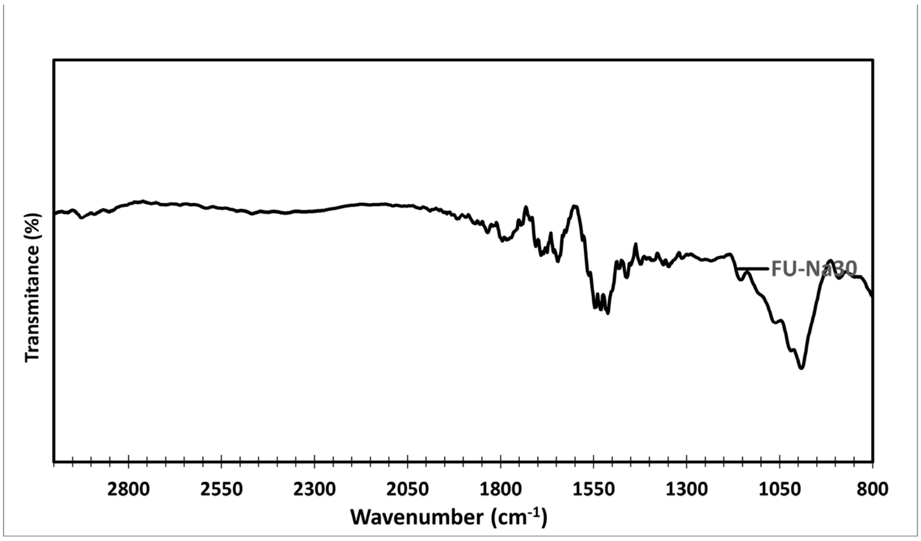

Figure S10. ATR-FTIR spectra of FU-Na30.

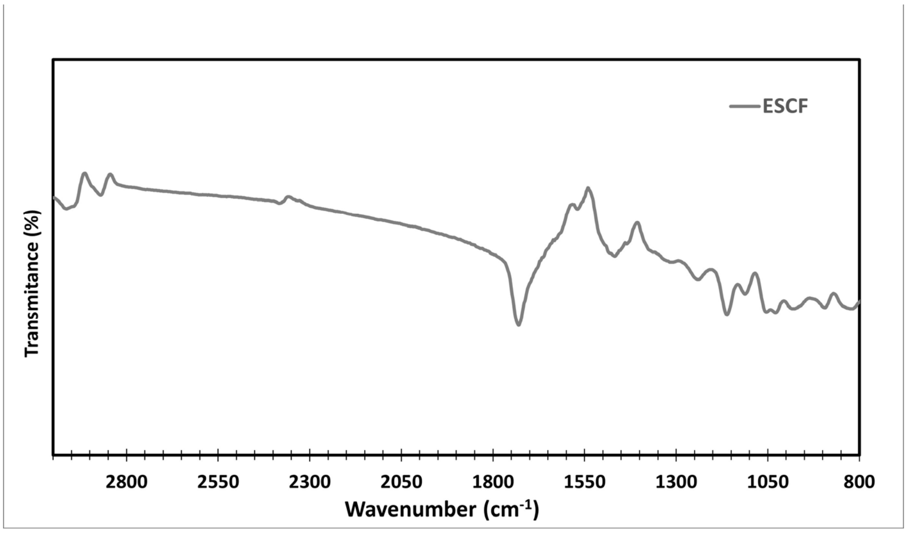

Figure S11. ATR-FTIR spectra of ESCF.

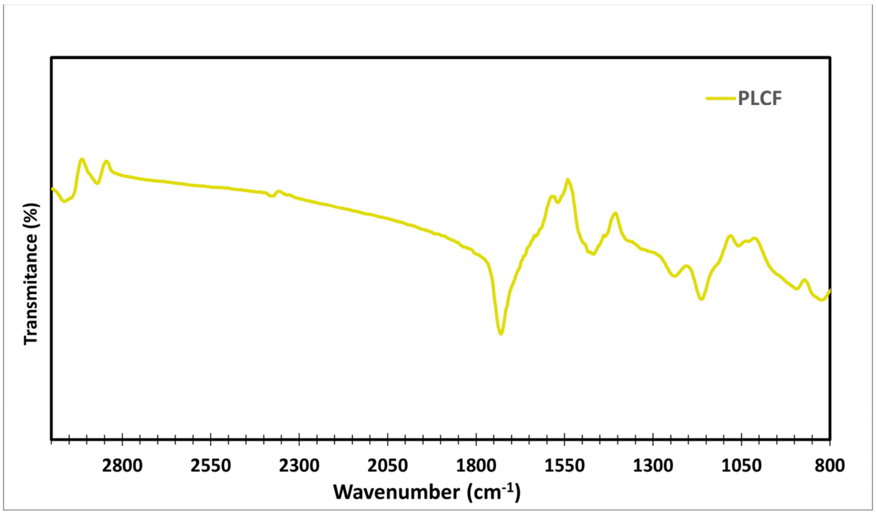

Figure S12. ATR-FTIR spectra of PLCF.
